# Foot kinematics in people with medial compartment knee osteoarthritis

**DOI:** 10.1186/1757-1146-4-S1-O27

**Published:** 2011-05-20

**Authors:** Pazit Levinger, Hylton B Menz, Adam D Morrow, Julian A Feller, John R Bartlett, Neil Bergman

**Affiliations:** 1 Musculoskeletal Research Centre, Faculty of Health Sciences, La Trobe University, Melbourne, Vic, 3086, Australia; 2 Warringal Medical Centre, Melbourne, Vic, 3084, Australia

## Background

Foot orthoses are commonly used in the management of knee osteoarthritis (OA), although the relationship between foot function and knee OA is still unclear. Therefore, the aim of this study was to compare tibial, rearfoot and forefoot motion during gait in people with and without medial compartment knee OA.

## Methods

Motion of the tibia, rearfoot and forefoot in 32 patients with clinically and radiographically-confirmed medial compartment knee OA (mean age 65.84 ± 7.57, height 168.83 ± 9.54 cm, body mass 85.13 ± 13.67 kg) and 28 age-matched controls (mean age 65.22 ± 11.41, height 168.61 ± 10.64cm, body mass 73.12 ± 15.49 kg) was investigated using a three dimensional motion analysis system incorporating a multisegment foot model (the Oxford Foot Model). Multivariate analysis was used to investigate the differences between the groups for peaks and ranges of motion with gait velocity entered as a covariate.

## Results

The knee OA group demonstrated greater peak rearfoot eversion (-3.8° ± 4.6 vs -0.7° ± 3.9; p < 0.001), contacted the ground with a more everted rearfoot at initial contact (0.6°± 5.4 vs 3.8° ± 3.7; p < 0.001) and exhibited reduced rearfoot frontal plane range of motion (8.6° ± 2.7 vs 10.4° ± 2.6; p = 0.02) and rearfoot peak inversion (4.8° ± 5.4 vs 9.6° ± 3.6; p < 0.001). The tibia was more internally rotated throughout the gait cycle with reduced range of motion (9.7° ± 4.2 vs 14.4° ± 4.0; p = 0.001) and peak external rotation compared to the control group (-20.1° ± 6.5 vs -27.6° ± 6.4; p = 0.002). Moreover, the tibia was tilted significantly more laterally in the knee OA group (7.8°± 3.4 vs 4.0° ± 1.9; p < 0.001) indicating a genu varum malalignment.

## Conclusions

People with medial compartment knee OA exhibit altered foot kinematics during gait that are indicative of a less mobile, flat foot deformity. Given that genu varum is a common feature in medial compartment knee OA, it is likely that the kinematic pattern observed occurs as a result of compensatory foot pronation to enable the foot to be plantigrade during gait.

